# Paths of cognitive and social-emotional delays before age three in rural China: Predictive power on skills at preschool age

**DOI:** 10.1371/journal.pone.0310016

**Published:** 2024-09-06

**Authors:** Lei Wang, Dingjing Jiang, Yifei Chen, Siqi Zhang, Scott Rozelle

**Affiliations:** 1 International Business School, Shaanxi Normal University, Shaanxi, China; 2 Stanford Center on China’s Economy and Institutions, Stanford University, Stanford, CA, United States of America; Aga Khan University Hospital, PAKISTAN

## Abstract

Cognitive and social-emotional development in the first three years of life is associated with later skills. However, little is known about the paths of developmental delays in both cognitive and social-emotional skills before age 3 or to what extent these paths predict later developmental outcomes. The aim of this study is to examine the associations between the different paths of developmental delays in both cognitive and social-emotional skills of children before age 3 and the levels of development of the children when they are preschool age. Using a longitudinal data collected at three time points from 1245 children and their caregivers in rural China, we identified four different paths of developmental delays in cognitive and social-emotional before age 3 and examined how these paths are associated with different levels of developmental outcomes at preschool age. We used a non-parametric standardization approach and an ordinary least squares model to perform our analyses. Findings show that rates of developmental delays in either cognitive or social-emotional domain or both domains are high at all different time points, ranging from 20% to 55% for cognitive delays and 42% to 61% for social-emotional delays. Over half of children experienced deteriorating levels of either cognitive or social-emotional development before age 3. A large share of children was found to be persistently delayed in either domain. Only a small share of children raised their levels of development in either domain before age 3. In addition, we identified certain socioeconomic status of the family that are associated with never or deteriorating path of child developmental delays. More importantly, we revealed that different paths of developmental delays before age 3 have predictive power on different levels of developmental outcomes at preschool age. Our results suggest that actions are needed at the earliest times to improve child development when children are still infants or toddlers.

## Introduction

It is well known that cognitive and social-emotional development in the first years of life are the foundations of the formation of later-life skills [1–4]. According to the literature, cognitive development refers to the mental process that improves ability of children to process information, including perceptual and linguistic processes, as well as conceptual, reasoning, and problem-solving [5–7]. Social-emotional development is defined as the process that helps children develop cooperative and pro-social behaviour, initiate and maintain peer friendships and adult relationships, manage aggression and conflict, develop a sense of mastery and self-worth and regulate emotions and reactivity [8]. Children before age 3 mostly depend on their relationships with parents or caregivers to teach them about themselves and the world they live in. Children who have developmental delays in the first three years, on average, have lower levels of development and school readiness when they reach to preschool age [9–13]. Studies also have highlighted the interplay between cognitive and social-emotional development starting from the very early years of life [14–18]. Delays in cognitive development before age three not only has been shown to affect future cognitive skills, but also often has negative effects on later-life social-emotional development [19–21]. Similarly, research shows that social-emotional delays in infanthood are negatively associated with both cognitive and social-emotional development at preschool age [22].

When examining the stability of cognition and social-emotional development, the literature has shown that both skills are quite dynamic before a child is three years old [23–30]. One study in the US found that 77% of children with mild and 70% of children with severe cognitive developmental delay at 9-months had no delay at 24-months. However, results of this study showed that children with cognitive delay at 9-months had 2.4 to 3 times the odds of having worse cognitive skills at 24-months compared to children with no cognitive delay at 9 months [29]. Another study, conducted in Finland, demonstrated that the prevalence of social-emotional delay of the children in the study’s sample decreased from 10% at 18-months to 5% at 36-months [26].

Since the developmental delays are dynamic before age 3, it would seem that it would be of great value to be able to identify the risk factors that are associated with the different paths of developmental delays. To our knowledge, however, only one study conducted in the US, has investigated the factors linked with the different paths of early childhood developmental delays. According to the study, child gender, birth weight, number of siblings of the child and the income of the family are all risk factors that lead to different paths of developmental delays [25]. Even though this study was conducted in the setting of a developed country, it is possible that these factors may also be associated with the different paths of early childhood developmental delays in developing settings.

Researchers also have focused on questions about how the paths of developmental delays when children are young affect the later skills of children as they age [25, 31–33]. According to these studies, different paths of developmental delays in earlier years have different associations with later skills. For example, a US study demonstrated that different paths of cognitive delays before the age of 3 induced different levels of severity of behavioural problems when the children were preschool age [25]. Children who experienced persistent delay, or those that were on an improving path, or those that were on deteriorating path of cognitive delays had more severe behavioural problems when they reached preschool age, comparing to children that had never been delayed in terms of cognitive skills before age 3. Dekker et al. [32] found that in the general population of Dutch citizens, aged 4 to 18, children who had followed a path of being persistently delayed in their social-emotional skills in late adolescence were most likely to have mental health problems and lower levels of education when they reached young adulthood. To the best of our knowledge, however, previous studies in this area have been focusing only on delays in one of child developmental domains, that is, delays in either cognitive or social-emotional skills while neglecting delays in the other domain.

Increasing the understanding of the predictive power of paths of developmental delays before age 3 on skills at preschool age should be of great importance in developing country settings where typically have relatively high rates of developmental delays in both the cognitive and social-emotional skills of young children [2, 34–36]. It is estimated that 219 million children (39%) under the age of 5 years old in low- and middle-income countries (LMICs) were not reaching their cognitive and/or social-emotional developmental potential [2]. Of these children in LMICs, it is estimated that there are 45 million under developed children are from rural China [37]. Studies in rural China have confirmed that the high prevalence of developmental delays of children under age 5 is a common problem across rural China; delay rates of cognitive skills were found to range from 39% to 49%; and delay rates of social-emotional skills were found to range from 34% to 58% [38–43].

Although previous studies in rural China have provided evidence that the rates of child developmental delays before age 3 are dynamic [38, 40, 41, 44], these studies have not identified the dynamic paths of cognition and social-emotional development. For example, Luo et al. [44] found that the rates of cognitive delay increased from 14% at 6 months to 49% at 29 months. However, the authors did not classify the different paths of cognitive delay of each child over the course of the study. Another study also found that the rates of cognitive delay were up to 55% at 30 months while they were 46% at 18 months; the rate of social-emotional development during these same times periods decreased from 56% at 18 months to 49% at 30 months [40]. Similarly, this study did not explore different paths of either cognitive delays or social-emotional delays of children between the ages of 18 to 30 months. In Wang et al. [41], even though used the same data set as the current study, the authors only identified the paths of social-emotional development of child before age 3 without considering the paths of child cognitive development simultaneously. The findings of the study by Wang et al. [41] missed scenarios: a) children who were delayed in both social-emotional and cognitive development; b) children who were not delayed in either domain of the development; and c) children who were delayed in one of domains while not delayed in the other. In this case, the findings of the study by Wang et al. [41] might underestimate the influence of the paths of child development during the period of age 0~3. Since the paths of dynamic development in other studies (in other countries) before the age of 3 are associated with the level of skills at preschool age [25, 31–33], it would be great value for China’s policymakers to understand the predictive power of different paths. Unfortunately, no study in rural China has provided such information.

The goal of the current study is to examine the association between the paths of developmental delays (both cognitive and social-emotional) in children before age 3 and their cognitive and social-emotional skills at preschool age in rural China. According to the literature, we expect that the sample children can be classified into four paths: (a) “never,” (b) “persistently,” (c) “improving,” or (d) “deteriorating.” We hypothesize that children that belong to the groups of children that have taken different paths will have different levels of cognitive and social-emotional skills at preschool age. We also expect that children in the “never” group will have higher levels of cognitive and social-emotional skills when they are preschool levels compared to other groups (i.e. persistently, improving, or deteriorating).

According to the results of the current study, rates of developmental delays in either cognitive or social-emotional development of child are high at all different time points before age 3. However, the delays are varying during the course of the study. In addition, the findings also show that different paths have different associations with later development when child are at preschool age. The findings suggest that identifying and addressing child developmental delays in either cognitive or social-emotional domain before age 3 may improve the levels of development at preschool age. This study adds to the literature by identifying the paths of both domains of child development before age 3 and investigating their predictive powers on skills at later childhood in rural China. To the best of our knowledge, this study is the first study to examine the paths of child development in cognitive and social-emotional domain simultaneously. We believe that the results of this study provide evidence to policymakers to implement interventions more accurately to improve both cognitive and social-emotional skills of rural children when they are still infants and toddlers.

The remainder of this paper is structured as follows. The Methods section presents our methods, including sample selection, data collection, and statistical methods. The Results section provides the results. The Discussion section presents a discussion of the findings and concludes.

## Methods

### Ethics approval statement

Approval for all data collection activities was obtained from the Stanford University Institutional Review Board (Protocol ID 25734). All participating caregivers gave their written informed consent for both their own and their infant’s involvement in the study. Participants were made aware of the risks involved and understood that their participation was purely voluntary. Specifically, upon arriving at each household during the data collection, the enumerators introduced the project and the interview and assessment procedure and asked the primary caregivers of the household if they agreed to participate the interview themselves and if they agreed their child to be accessed. After receiving their consent, the caregivers proceeded to sign the provided consent forms. All methods in this study were carried out in accordance with relevant guidelines and regulations.

### Study design

Data were drawn from a longitudinal study conducted by authors in 11 nationally-designated poverty counties in western China. In 2013, 2015 and 2017, research team collected data that were used for this study.

### Sampling and participants

The sample children were recruited in 2013 when they were 6–12 months old. The sample selection followed a multistage cluster design. First, all townships in the 11 counties were included and the total number of townships was 174. After the selection of the township, two villages from each township were randomly selected. Finally, all children within target age range (6–12 months) in each selected village were included in this study. Following the initial recruitment, the second and the third surveys were conducted in April 2015 when sample children were 22–30 months old and in 2017 when sample children were aged 49–65 months, respectively. The total sample in this study included 1245 children (51.4% Male, 48.6% Female) and their primary caregivers who participated all three waves of the surveys implemented in 2013, 2015 and 2017. All of the sample participants were Han ethnicity and lived in rural villages.

Table 1 describes the demographics of the sample children, caregivers and their families. Among the sample children, only a small portion of children (4.7%) were born prematurely. More than three quarters (76.1%) of the sample children were the only child of the household (that is, they had no siblings). For 85% of the children, the mother was the primary caregiver. Among the mothers, 61.8% were more than 25 years old, and 72% had obtained at least 9 years of schooling.

**Table 1:** Characteristics of sample children (6–12 months).

Characteristic	Frequency (*n*)	Percentage (*%*)
*Child*		
Gender		
Male	640	51.4
Female	605	48.6
Whether the child was premature		
Yes	58	4.7
No	1,187	95.3
Whether the child has siblings		
Yes	298	23.9
No	947	76.1
*Household*		
Mother is primary caregiver		
Yes	1,058	85.0
No	187	15.0
Maternal age		
< 25	476	38.2
≥ 25	769	61.8
Maternal education level (years)		
< 12	1057	84.9
≥ 12	188	15.1
Family asset index		
Top tercile	356	28.6
Middle tercile	387	31.1
Bottom tercile	502	40.3

### Measures

#### Child cognitive development

Two measures were used in this study to assess child cognitive development at different age. When the sample children were 6–12 months and 22–30 months (i.e., in the first and second survey waves), the cognitive development of each child was assessed by using the first version of the Bayley Scales of Infant Development (BSID). At the time of our survey, this version of the BSID was the only version of the test adapted to the Chinese language and cultural environment, and it had been used in a number of child development studies throughout China [45–49]. The BSID is a gold standard test for measuring child cognitive development under 30 months of age [50, 51]. The test was formally adapted to the Chinese language and settings and scaled according to an urban Chinese sample [52]. The BSID produces a measure of cognitive development: the mental developmental index (MDI), which measures memory, habitation, problem solving, early number concepts, generalization, classification, vocalizations, and language [53]. The MDI has an expected mean of 100 and standard deviation (SD) of 16 in the Chinese version [52]. In this study, children who had an MDI score below 84 (1 SD below the mean) are considered to be delayed in cognitive development.

When the sample children were 49–65 months old, the cognitive development of the sample children was assessed by the Wechsler Preschool and Primary Scale of Intelligence-Fourth Edition (WPPSI-IV), which was the latest version of the scale that was translated into Chinese and had a Chinese norm at the time of our survey. The WPPSI-IV is still the most updated and most widely used instrument that researchers have used to assess the cognitive development of preschool children in mainland China [54–56]. The WPPSI-IV is an internationally recognized and standardized test for assessing the cognitive development of children aged 30–91 months [57]. In 2010, The WPPSI-IV was translated and adapted into a Chinese version and scaled according to a sample from China containing participants from both urban and rural areas [58]. The WPPSI-IV produces a Full-Scale Intelligence Quotient (FSIQ), which is a composite score that summarizes the subject’s cognitive ability across a diverse set of domains. In this study, children who had FSIQ scores below 85 (1 SD below the mean) were considered to be delayed in cognitive development [58].

#### Child social-emotional development

In all three survey waves, the measure of the Ages and Stages Questionnaire: Social-Emotional (ASQ:SE) was used to assess the social-emotional development skills of each child. The ASQ:SE is a validated and widely used tool to measure social-emotional development of children aged 3–66 months [59]. In 2013, the ASQ:SE was translated into Chinese and researchers began to use this version in China [60–62]. In 2017, the ASQ:SE was modified and validated in China. The ASQ:SE includes different sets of items to assess child social-emotional development at different age, including the ability of the child to calm down, the propensity of the sample individuals to be able to accept directions, the ability of the child to demonstrate feelings towards others, skills for communicating his/her feelings as well as others (e.g., the ability to initiate social responses to parents/others and how to rejoin on one’s own initiative). During each survey, the primary caregiver of each sample child was asked to rate whether the child exhibits these behaviors “most of the time,” “sometimes,” or “never”, and answers were scored 0, 5, or 10 points. The Manual of the ASQ:SE provides the cutoffs for different age ranges to identify if the child is at risk for having social-emotional problems (S1 Table). These cutoffs were used to define the delays of the sample children in social-emotional development in each wave. Specifically, children who had ASQ:SE scores higher than the cutoff set for different ages are considered to be at risk of delay in social-emotional development.

### Procedures

At each time point, students from local universities were recruited and conducted data collection. The students were divided into teams of enumerators and teams of testers. Enumerators collected socioeconomic and child social-emotional development data by interviewing the primary caregivers. Testers were in charge of the assessment of child cognitive development. Prior to each survey wave, the enumerators received a three-day-long training course on basic theory of early childhood development (ECD), survey administration, and administration of the Bayley Scales of Infant Development. When arriving at each household during each survey wave, trained enumerators first identified the primary caregiver as the individual most responsible for the child’s daily care (typically the child’s mother or paternal grandmother). The primary caregiver was then administered a detailed survey that elicited information on their own characteristics, their child’s characteristics and the characteristics of the household, including the child’s age and gender, whether the child was born premature, whether the child had siblings, the caregiver’s age and educational level, and the family’s assets. Using the information on the family’s assets, the research team constructed a household assets index using a polychronic principal component approach. The asset index included the following variables: tap water, toilet, water heater, washing machine, computer, internet, refrigerator, air conditioner, motor or electronic bicycle, and car. During each survey, the primary caregivers also were asked to assess the social-emotional development of the child by rating the items in the ASQ:SE based on the age of their children.

Child cognitive development was assessed in all survey waves using standard measures based on the Bayley Scale of Infant Development approach for the children when they were under three years old and the Wechsler Preschool and Primary Scale of Intelligence for the children when they were 4 to 5 years old in preschool. Prior each survey wave, the testers underwent a formal week-long training course, including 2.5 days of field training. The assessments were administered one on one, either at home or in the child’s preschool. Neither caregivers nor teachers were allowed to assist children during the assessments.

### Statistical analyses

Following the literature [25, 63], the sample children were classified into four path groups based on whether they were delayed in development over the course of infancy (6–12 months) to toddlerhood (22–30 months). The four groups are (a) “Never delayed”–children had no delays in either cognitive or social-emotional development over the course of infancy to toddlerhood; (b) “Persistently delayed”–children had delays in either or both domains of development in infancy and still had delays in either or both domains in toddlerhood; (c) “Improving”–children had delays in either or both domains in infancy and only had delays in one of two domains or no longer had delays in either domain at toddlerhood; (d) “Deteriorating”–children had no delays in either domain or had delays only in one of two domains in infancy but had delays in either or both domains in toddlerhood.

Next, using the non-parametric standardization method developed by Rubio et al. [64], MDI raw scores, FSIQ raw scores, and ASQ:SE raw scores were internally standardized separately for each survey wave. The internally standardized process refers to the process of standardizing the raw scores of each measurement within each age-month group. To be specific, age-conditional means and standard deviations for each age-month group were estimated by using the non-parametric regression method (detailed information can be found in Rubio et al., 2016). These estimated statistics were then used to compute age-adjusted internal standardized scores to eliminate the age effect since all the raw scores of each scale are increasing in age.

Finally, an ordinary least squares (OLS) model was constructed to estimate the associations between different path groups (from the time children were infants until they were toddlers) and the development outcomes (cognitive and/or social-emotional development) of children at preschool age. In this analysis, when we examine the associations between the never delayed group and child development at preschool age, we take other three groups as references. In contrast, when we examine the associations between the persistently delayed group or the improving group or the deteriorating group with child development at preschool age, we take the never group as a reference group. We also used the same OLS approach with an alternative specification to estimate the associations between the paths and child development at preschool age. In this analysis, we put all groups separately into one model, taking the never delayed group as a reference group.

## Results

### Child developmental outcomes

Table 2 presents the cognitive and social-emotional scores, rates of cognitive and social-emotional delays of sample children at three time points (6–12 months, 22–30 months and 49–65 months), by reporting means and standard deviations. Regardless of the different measures of child cognitive development at different time points (i.e. MDI and FSIQ), the mean scores were all below the mean of 100 that have been shown to be the normed average of healthy populations. When children were 6–12 months old, 20% of them were found to be cognitively delayed. The share of the cognitive delay increased to 55% at 22–30 months old and was still relatively high rate (45%) when children were 49–64 months old.

**Table 2:** Cognitive and social-emotional developmental outcomes (N = 1,245).

	Infancy	Toddlerhood	Preschool age
	Mean (SD)	Mean (SD)	Mean (SD)
	(1)	(2)	(3)
Cognitive scores	96.25 (16.70)	81.01 (21.49)	88.67 (11.75)
Rate of cognitive delay	0.20 (0.40)	0.55 (0.50)	0.45 (0.50)
Social-emotional scores	45.75 (26.94)	71.18 (39.13)	81.10 (45.60)
Rate of social-emotional delay	0.42 (0.49)	0.61 (0.49)	0.52 (0.50)

*Note*. Cognitive scores are the Bayley Scale of Infant Development (BSID) scores on the Mental Development Index (MDI) for infants (6–12 months) and toddlers (22–30 months), and the Wechsler Preschool and Primary Scale of Intelligence-Fourth Edition (WPPSI-IV) scores on the Full-Scale Intelligence Quotient (FSIQ) for preschool-age children (49–65 months). Social-emotional scores are the scores of Ages & Stages Questionnaires: Social-Emotional (ASQ:SE). The higher the score, the worse the social-emotional development.

Table 2 also includes the scores at all three time points from the ASQ:SE that is a measure of the social-emotional development of the sample children. The mean scores of the ASQ:SE scales at each point of time were all higher than the cutoffs set by the ASQ:SE Manual for the respective age ranges (see S1 Table). The results of the ASQ:SE analysis demonstrate that a significant share of children experienced social-emotional delay over the course of infancy through toddlerhood to preschool age.

### Paths of child developmental delays from infancy to toddlerhood

According to the definitions of the paths of developmental delays proposed in Methods Section, the sample children were classified into four paths of developmental delays from infancy (6–12 months) to toddlerhood (22–30 months). Table 3 presents the distributions of these four paths among sample children by reporting the frequencies and percentages of each path. Of the 1,245 children in the full sample, over half (n = 660; 53%) were suffering deteriorating development either in cognitive ability or in social-emotional skills. The analysis also shows that 301 (24.2%) were persistently delayed in either or both of the two domains of development. In the sample, only 141 children (or 11.3% of the sample) were never delayed and only 143 children (or 11.5% of the sample) showed improving levels of development in either domain over the course of infancy to toddlerhood. S2 Table provides more detail information on the definitions and distributions of the four paths of developmental delays.

**Table 3:** Paths of child cognitive and social-emotional delays from infancy to toddlerhood.

	Frequency (n)	Percentage (%)
Never delayed	141	11.3
Persistently delayed	301	24.2
Improving	143	11.5
Deteriorating	660	53.0

#### Association between demographic characteristics and paths of developmental delays from infancy to toddlerhood

Table 4 presents multivariate logistic estimates of associations between child/household characteristics and the paths of child cognitive and social-emotional delays from infancy to toddlerhood, using multivariate logistic regression model. The results show that certain demographic characteristics are significantly associated with different paths of child developmental delays. Specifically, boys were less likely to be in the group of never delayed compared to girls (Odds Ratio, OR = 0.70, p < 0.05). If born prematurely, the child was more likely to improve in either or both cognitive or social-emotional delays during the course of infancy to toddlerhood (OR = 2.25, p < 0.05). For those children whose mothers had attained at least 12 years of schooling, they were more likely not to have experienced any delays (i.e., following the never delay path) in either cognitive or social-emotional skills (OR = 1.75, p < 0.05); children of mothers with at least 12 years of schooling also were less likely to have been on the deteriorating development path (OR = 0.69, p < 0.05), compared to their peers whose mothers had less than 12 years of education. Finally, the results also indicate that children from households in the middle (OR = 1.46, p < 0.05) or bottom terciles (OR = 1.60, p < 0.01) of family assets were more likely to suffer deteriorating development; children from households in the bottom tercile of family assets were also less likely to have been on the never delayed path (OR = 0.55, p < 0.05), relative to children from households in the top tercile of family assets.

**Table 4:** Associations between demographic characteristics and paths of child developmental delays (N = 1,245).

	Never delayed		Persistently delayed		Improving		Deteriorating	
	OR	95% CI	OR	95% CI	OR	95% CI	OR	95% CI
Gender								
Female	Ref.		Ref.		Ref.		Ref.	
Male	0.70**	(0.49, 0.98)	1.20	(0.91, 1.58)	0.97	(0.68, 1.39)	1.02	(0.81, 1.30)
Born prematurely								
No	Ref.		Ref.		Ref.		Ref.	
Yes	1.25	(0.57, 2.74)	0.92	(0.47, 1.80)	2.25**	(1.10, 4.58)	0.61	(0.33, 1.14)
Has siblings								
No	Ref.		Ref.		Ref.		Ref.	
Yes	1.07	(0.69, 1.67)	1.18	(0.84, 1.65)	1.12	(0.75, 1.66)	0.82	(0.60, 1.11)
Mother is primary caregiver								
No	Ref.		Ref.		Ref.		Ref.	
Yes	1.48	(0.84, 2.60)	0.89	(0.62, 1.27)	0.87	(0.53, 1.43)	1.00	(0.72, 1.41)
Maternal age								
< 25	Ref.		Ref.		Ref.		Ref.	
≥ 25	1.16	(0.77, 1.74)	0.99	(0.75, 1.32)	1.50	(1.00, 2.26)	0.81	(0.63, 1.04)
Maternal education level (years)								
< 12	Ref.		Ref.		Ref.		Ref.	
≥ 12	1.75**	(1.09, 2.81)	1.06	(0.72, 1.56)	1.12	(0.65, 1.92)	0.69**	(0.49, 0.98)
Family asset index								
Top tercile	Ref.		Ref.		Ref.		Ref.	
Middle tercile	0.64	(0.41, 1.01)	0.98	(0.68, 1.40)	0.69	(0.42, 1.13)	1.46**	(1.07, 2.01)
Bottom tercile	0.55**	(0.35, 0.89)	0.81	(0.56, 1.16)	0.89	(0.55, 1.45)	1.60***	(1.17, 2.20)

*p < .1

**p < .05

***p < .01

#### Association between demographic characteristics and paths of developmental delays from infancy to toddlerhood

Tables 5 and 6 present the associations between paths of developmental delays before age 3 and the child developmental outcomes of the sample children when they were preschool age, using OLS with two alternative approaches. Table 5 compares developmental scores of children in each individual path of developmental delays to the children in the other three delay paths. As seen in columns (1) and (5), children that were never delayed from infancy to toddlerhood had significantly higher levels of cognitive and social-emotional development when they were preschool age when they were compared to children in the other three delay paths. In contrast, children suffered deteriorating cognitive abilities and/or deteriorating social-emotional development skills over the course of infancy to toddlerhood had significantly lower levels of cognitive and social-emotional development when they were preschool-age relative to the rest of sample children (columns 4 and 8). Finally, children that improved in development between infancy to toddlerhood had significantly higher levels of social-emotional development when they were preschool age when they were compared to children in the other three delay path groups (column 7). However, no significant association was found between improving path and child cognitive development at preschool age. In addition, no significant associations were found between persistently path and child cognitive or social-emotional outcomes at preschool age.

**Table 5:** Associations between cognitive and social-emotional scores at preschool age and different paths of child cognitive and social-emotional delays from infancy to toddlerhood (N = 1,245).

	Standardized FSIQ scores (at preschool age)				Standardized ASQ:SE scores (at preschool age)			
	(1)	(2)	(3)	(4)	(5)	(6)	(7)	(8)
Never delayed	0.35***				-0.34***			
(1 = Never, 0 = Otherwise)	(0.08)				(0.08)			
Persistently delayed		-0.08				0.11		
(1 = Persistently, 0 = Otherwise)		(0.07)				(0.07)		
Improving			0.13				-0.18**	
(1 = Improving, 0 = Otherwise)			(0.09)				(0.08)	
Deteriorating				-0.14***				0.13**
(1 = Deteriorating, 0 = Otherwise)				(0.06)				(0.06)
Control variables	Yes	Yes	Yes	Yes	Yes	Yes	Yes	Yes
County fixed effect	Yes	Yes	Yes	Yes	Yes	Yes	Yes	Yes
Time fixed effect	Yes	Yes	Yes	Yes	Yes	Yes	Yes	Yes

*Note*. Each column is a separate regression. Control variables include child’s age, gender, whether the child premature, whether the child has siblings, whether the mother is the primary caregiver, whether maternal age is above 25 years old, whether the mother has attained more than 12 years of education, and family asset index. We also control for time and county fixed effect. All standard errors account for clustering at the village level. *p < .1

**p < .05

***p < .01

**Table 6:** Associations between cognitive and social-emotional score at preschool age and paths of child cognitive and social-emotional delays from infancy to toddlerhood (*N* = 1,245).

	Standardized FSIQ scores (at preschool age)	Standardized ASQ:SE scores (at preschool age)
	(2)	(4)
Persistently	-0.38***	0.38***
(1 = Persistently, 0 = Otherwise)	(0.10)	(0.09)
Improving	-0.20	0.14
(1 = Improving, 0 = Otherwise)	(0.12)	(0.10)
Deteriorating	-0.38***	0.36***
(1 = Deteriorating, 0 = Otherwise)	(0.09)	(0.08)
Control variables	Yes	Yes
County fixed effect	Yes	Yes
Time fixed effect	Yes	Yes

*Note*. Each column is a separate regression. “Never delayed” group was taken as a reference group in the regressions. Control variables include child’s age, gender, whether the child premature, whether the child has siblings, whether the mother is the primary caregiver, whether maternal age is above 25 years old, whether the mother has attained more than 12 years of education, and family asset index. We also control for time and county fixed effect. All standard errors account for clustering at the village level. *p < .1

**p < .05

***p < .01

Table 6 shows the estimates of the associations between paths of developmental delays before age 3 and the levels of development at preschool age using an alternative method. In this analysis, we took the never delayed children as a reference group. The results demonstrate that children in all of the other three paths (i.e., persistently delayed, improving, or deteriorating) had significantly lower cognitive scores at preschool compared to children that were never delayed before age 3. In the case of social-emotional development, children that were persistently delayed or had deteriorating levels of development before age 3 had significantly lower levels of social-emotional development at preschool compared to children that were never delayed before age 3. Children with improving development over the course of infancy and toddlerhood, however, demonstrated no significant differences in social-emotional development compared to children that were never delayed.

## Discussion

Although there have been studies on the associations between the paths of delays in one of the child developmental domains (either cognitive or social-emotional skill) before age 3 and later developmental outcomes [25, 31–33], additional research is needed to understand such associations in the settings of developing countries, considering delays in both cognitive and social-emotional skills simultaneously. In this study, we identified the paths of child cognitive and social-emotional delays before age 3 in rural China and examined the predictive powers of these development paths (before age 3) on the child development outcomes at preschool age.

The results of the analysis of this study show that rates of developmental delays in either domain (cognitive or social-emotional skill) are relatively high at all different time points in the study (which examines children between those ages of 6 months and preschool age), ranging from 20% to 55% for cognitive delays and 42% to 61% for social-emotional delays. The findings are consistent with previous studies in rural China which have found high prevalence of early childhood developmental delays in rural China [38–43]. Our findings, along with previous studies, indicate that the existence of high levels of developmental delays among rural children in early childhood is a common problem across rural China. The reason of this problem, according to the results of previous studies in rural China, may lie in the low levels of parental parenting beliefs and practices [38, 40, 65–67].

The findings of this study also demonstrate that the developmental delays are not constant during the course of the study. Over half of the children in our sample experienced deteriorating levels of either cognitive or social-emotional development before age 3; a large share of children was found to be persistently delayed in either cognitive or social-emotional skills. Importantly, only a small share of the sample children raised their levels of development in either domain before age 3. Such findings are in line with previous studies in rural China [41, 44]. The findings suggest strongly that actions are needed at the earliest times to improve the development when children are still infants/toddlers. If action is not taken, the findings illustrate that the low levels of developmental outcomes will be persistent or even worsen through the course of the child’s life between infanthood to preschool age.

In addition, we identified certain demographic characteristics (that is, the socioeconomic status, or SES, of the rural family) that are associated with never or deteriorating paths of child developmental delays. More educated mothers and relatively rich rural families are more likely to have children who have not experienced any delays (or are in the “never” category) in either cognitive or social-emotional delays before age 3. In contrast, less educated mothers and relatively poor rural families are more likely to have children who have suffered deteriorating developmental outcomes before age 3. These findings are in line with a previous study that has shown the SES is a risk factor associated with the different paths of early childhood development [25]. The underlying reason for this may be that caregivers from families with relatively high levels of SES are more likely to know better about the importance of early childhood development, leading more involvement in the child development and thus improving child developmental outcomes [41, 44]. In contrast, caregivers from a relatively low levels of SES may not understand the importance of early childhood development and thus be less involved in the development of their children, which may be part of the reason that leads to their children’s status as having persistent or deteriorating developmental delays before age of 3. To be clear, we also examined the associations between demographic characteristics with two other paths, persistently delayed and improving; however, no significant associations were found.

More importantly, the findings of our study reveal that different paths of developmental delays before age 3 have predictive power on different levels of developmental outcomes (including both cognitive and social-emotional skills) at preschool age. Even though there has been no study examining delays in both domains simultaneously, these findings are in line with previous studies that investigated the associations between developmental delays in either one of the two domains before age 3 and developmental outcomes at preschool age [21, 25, 31, 32]. The findings of this study that children on the never and improving paths had similar levels of cognitive and social-emotional development at preschool age reveal that identifying and addressing developmental delays in either domain before age 3 may improve the developmental levels at preschool age. Otherwise, children would suffer persistently delays or deteriorating development not only before age 3 but also have low levels of development in later life. The underlying reason of such findings may lie in that, on one hand, the foundations of the skills of later life are mainly formed in the first three years of life [2]. Children who are delayed in development won’t have capability to develop well in the future [9–11]. On the other hand, cognitive and social-emotional development of child have the interplay starting from early age and such interplay persists in a longer term [15–17]. The lower levels of either domain of development affect both domains of development in the future [19–21].

The results of this study have several implications for policymakers. Considering the high rates of cognitive and social-emotional delays before age 3, and the predictive powers of the different paths of developmental delays on developmental outcomes at preschool age in rural China, policy makers need to take actions to improve the developmental outcomes of rural children at an early age (when the children are infants/toddlers). The findings that higher SES families are more likely to have children that are never delayed may help policy makers to implement an intervention aiming to enhance the perception of rural families about the importance of early childhood development, leading to improve parental involvement in child development and thus achieve higher levels of child development among the rural population. These findings suggest several approaches that might be used to carry out such interventions to the public. Information campaigns in the media that convey messages of the importance of parenting as well as efforts by the medical personnel (during prenatal visits) that encourage parents to be more involved in psycho-stimulating interactions with their babies may be two ways to raise awareness. Parenting training programs may be another effective way to instruct rural parents to perform age-appropriate interactions with their children. A systematic review study of the randomized controlled trials conducted in rural China has confirmed that such program can have significant effects on parenting practices and child development [38].

This study makes three contributions to the literature. First, data used in this study were drew from a longitudinal survey with a large sample size across hundreds of villages in rural China, which enhances the validity and representativeness of the findings. Second, even though it is well-known that the development of children before age 3 can have short- and long-run effects on the level of development in later life, it is not clear what the different paths of the development before age 3 are, and whether these different paths have different effects on the later life outcomes of the children. This is the first study that has investigated paths of development both in cognitive and social-emotional skills simultaneously and the associations between these paths and the developmental outcomes of children when they are preschool age in rural China. The findings fill the gap in the literature on the different paths of both cognitive and social-emotional developmental delays before age 3 and their predictive powers on skills at preschool age in rural China. Finally, this study identified factors linked with different paths of developmental delays before age 3, findings that can help policy makers to more accurately target the children that are most vulnerable and are most likely to be on the paths of either persistently delayed or deteriorating delays. With such information, it is hoped that policy makers can design and implement interventions that consider those factors that are known to influence the different paths in order to improve both cognitive and social-emotional skills of rural children when they are still infants and toddlers.

While this study has a number of strengths, there still are limitations. First, even though we investigated child developmental outcomes at three different points of time, it is possible that there are still other critical points of time that we missed that may also matter in the development of the sample children. In this case, we may have under-estimated the rates of cognitive or social-emotional delays. Second, although the families/caregivers/children in the sample that were involved in this study were randomly selected across a large number of villages, we do not consider the findings of this study to be representative of rural China as a whole. Future research works need to continue to study these issues in other rural areas across the whole nation. Third, even though this study uses longitudinal data, the findings based on the OLS estimates cannot be interpreted as being causal inferences. Further research is needed to explore the causal effects in this area.

## Supporting information

S1 Table Applicable scope and cutoff score of each ASQ: SE questionnaire interval set by the manual of the ASQ: SE. (DOCX)

S2 Table Definitions and distributions of paths of developmental delays from infancy to toddlerhood. (DOCX)
