# Supplementary material for: Paths of cognitive and social-emotional delays before age three in rural China: Predictive power on skills at preschool age
Source: PLoS One. 2024 Sep 6;19(9):e0310016. doi: 10.1371/journal.pone.0310016 (PMC11379282; doi:10.1371/journal.pone.0310016)
Supplement: S1 Table — (DOCX) [file pone.0310016.s001.docx]

**S1 Table. Applicable scope and cutoff score of each ASQ:SE questionnaire interval set by the Manual of the ASQ: SE.**

| Questionnaire interval | Applicable range | Cutoff score |
| --- | --- | --- |
| 6 months | 3–8 months | 45 |
| 12 months | 9–14 months | 48 |
| 18 months | 15–20 months | 50 |
| 24 months | 21–26 months | 50 |
| 30 months | 27–32 months | 57 |
| 36 months | 33–41 months | 59 |
| 48 months | 42–53 months | 70 |
| 60 months | 54–65 months | 70 |
