# Supplementary material for: Paths of cognitive and social-emotional delays before age three in rural China: Predictive power on skills at preschool age
Source: PLoS One. 2024 Sep 6;19(9):e0310016. doi: 10.1371/journal.pone.0310016 (PMC11379282; doi:10.1371/journal.pone.0310016)
Supplement: S2 Table — (DOCX) [file pone.0310016.s002.docx]

**S2 Table. Definitions and distributions of paths of developmental delays from infancy to toddlerhood.**

|  | Infancy | | Toddlerhood | | Frequency  (n) | Percentage  (%) |
| --- | --- | --- | --- | --- | --- | --- |
|  | Cognition | Social-emotion | Cognition | Social-emotion |  |  |
| Never delayed | No | No | No | No | 141 | 11.3 |
| Persistently delayed | Yes | Yes | Yes | Yes | 58 | 4.7 |
|  | Yes | No | Yes | No | 41 | 3.3 |
|  | Yes | No | No | Yes | 27 | 2.2 |
|  | No | Yes | No | Yes | 124 | 10.0 |
|  | No | Yes | Yes | No | 51 | 4.1 |
| Total of Persistently delayed | | | | | 301 | 24.2 |
| Improving | Yes | Yes | No | No | 11 | 0.9 |
|  | Yes | Yes | No | Yes | 22 | 1.8 |
|  | Yes | Yes | Yes | No | 29 | 2.3 |
|  | Yes | No | No | No | 23 | 1.8 |
|  | No | Yes | No | No | 58 | 4.7 |
| Total of improving | | | | | 143 | 11.5 |
| Deteriorating | No | No | Yes | Yes | 170 | 13.7 |
|  | No | No | Yes | No | 126 | 10.1 |
|  | No | No | No | Yes | 158 | 12.7 |
|  | No | Yes | Yes | Yes | 169 | 13.6 |
|  | Yes | No | Yes | Yes | 37 | 3.0 |
| Total of deteriorating | | | | | 660 | 53.0 |
| Full sample | | | | | 1,245 | 100 |
